# Infections in temporal proximity to HPV vaccination and adverse effects following vaccination in Denmark: A nationwide register-based cohort study and case-crossover analysis

**DOI:** 10.1371/journal.pmed.1003768

**Published:** 2021-09-08

**Authors:** Lene Wulff Krogsgaard, Irene Petersen, Oleguer Plana-Ripoll, Bodil Hammer Bech, Tina Hovgaard Lützen, Reimar Wernich Thomsen, Dorte Rytter

**Affiliations:** 1 Research Unit for Epidemiology, Department of Public Health, Aarhus University, Aarhus C, Denmark; 2 Department of Clinical Epidemiology, Aarhus University Hospital, Aarhus N, Denmark; 3 Department of Primary Care and Population Health, Institute of Epidemiology and Health Care, Faculty of Population Health Sciences, University College London, United Kingdom; 4 National Centre for Register-based Research, Aarhus University, Aarhus V, Denmark; Universitair Medisch Centrum Utrecht, NETHERLANDS

## Abstract

**Background:**

Public trust in the human papilloma virus (HPV) vaccination programme has been challenged by reports of potential severe adverse effects. The reported adverse symptoms were heterogeneous and overlapping with those characterised as chronic fatigue syndrome (CFS) and have been described as CFS-like symptoms. Evidence suggests that CFS is often precipitated by an infection. The aim of the study was to examine if an infection in temporal proximity to HPV vaccination is a risk factor for suspected adverse effects following HPV vaccination.

**Methods and findings:**

The study was a nationwide register-based cohort study and case-crossover analysis. The study population consisted of all HPV vaccinated females living in Denmark, born between 1974 and 2006, and vaccinated between January 1, 2006 and December 31, 2017. The exposure was any infection in the period ± 1 month around time of first HPV vaccination and was defined as (1) hospital-treated infection; (2) redemption of anti-infective medication; or (3) having a rapid streptococcal test done at the general practitioner. The outcome was referral to a specialised hospital setting (5 national HPV centres opened June 1, 2015) due to suspected adverse effects following HPV vaccination. Multivariable logistic regression was used to estimate the association between infection and later HPV centre referral. The participants were 600,400 HPV-vaccinated females aged 11 to 44 years. Of these, 48,361 (9.7%) females had a hospital-treated infection, redeemed anti-infective medication, or had a rapid streptococcal test ± 1 month around time of first HPV vaccination. A total of 1,755 (0.3%) females were referred to an HPV centre. Having a hospital-treated infection in temporal proximity to vaccination was associated with significantly elevated risk of later referral to an HPV centre (odds ratio (OR) 2.75, 95% confidence interval (CI) 1.72 to 4.40; *P* < 0.001). Increased risk was also observed among females who redeemed anti-infective medication (OR 1.56, 95% CI 1.33 to 1.83; *P* < 0.001) or had a rapid streptococcal test (OR 1.45, 95% CI 1.10 to 1.93; *P* = 0.010). Results from a case-crossover analysis, which was performed to adjust for potential unmeasured confounding, supported the findings. A key limitation of the study is that the HPV centres did not open until June 1, 2015, which may have led to an underestimation of the risk of suspected adverse effects, but stratified analyses by year of vaccination yielded similar results.

**Conclusions:**

Treated infection in temporal proximity to HPV vaccination is associated with increased risk for later referral with suspected adverse vaccine effects. Thus, the infection could potentially be a trigger of the CFS-like symptoms in a subset of the referred females. To our knowledge, the study is the first to investigate the role of infection in the development of suspected adverse effects after HPV vaccination and replication of these findings are needed in other studies.

## Abstract 2

Lene Wulff Krogsgaard and co-workers study temporal associations between infections and HPV vaccination.

## Author summary

**Why was this study done?:**

- In Denmark, there was a human papilloma virus (HPV) vaccination crisis in 2015, where reports of suspected adverse effects after HPV vaccination started increasing and the HPV vaccination uptake decreased dramatically.
- All available evidence has found the HPV vaccine to be safe and the specific cause of the reported adverse effects remains unknown.
- The reported symptoms were overlapping with those characterised as chronic fatigue syndrome (CFS) and have been described as CFS-like.
- Infections are thought to be triggers of CFS, and we hypothesise that infections in temporal proximity to vaccination could also play a role for the development of suspected adverse effects following HPV vaccination.

**What did the researchers find?:**

- Females who had a treated infection in temporal proximity to HPV vaccination were more likely to be referred to a specialised hospital setting (HPV centre) because of suspected adverse effects after HPV vaccination compared to HPV-vaccinated females without an infection. The case-crossover and case time-control analyses made to control for potential unmeasured confounding supported this finding.

**What do these findings mean?:**

- Infections might be a trigger of the suspected adverse effects in a subset of females referred to an HPV centre.
- More research is needed in order to confirm these findings and to clarify whether infections alone or in combination with HPV vaccine could have caused the reported symptoms.

## Introduction

Human papilloma virus (HPV) vaccination has been shown to prevent cervical intraepithelial neoplasia, which is preexisting to cervical cancer [1–3]. The HPV vaccination was introduced as a self-paid vaccination in Denmark in 2006. Following this, a start-up programme was initiated in 2008, and the HPV vaccine was implemented in the Danish childhood vaccination programme in 2009 targeting 12-year-old girls. In addition, several catch-up programmes have been completed, and, presently, all females born after 1984 have been offered the HPV vaccine at some point. Initially, the HPV vaccine had great support with a vaccination uptake above 90% [4]. However, in 2015, the vaccination uptake decreased substantially because of an increasing number of reported adverse effects resulting in public safety concerns [5]. Consequently, in June 2015, the Danish government established 5 specialised hospital settings (HPV centres) in order to medically evaluate and treat females with suspected adverse effects following vaccination. The most frequently reported symptoms were malaise, fatigue, dizziness, pain, and cognitive impairment [6]. However, no available evidence has found a causal link between HPV vaccination and the reported symptoms and diagnoses [7–15]. However, most of the existing evidence is based on studies investigating specific hospital discharge diagnosis, which might not capture all females with suspected adverse effects, e.g., if the females are predominantly seen in primary care, emergency rooms, or outpatient clinics [8–11,15,16].Some of the females reported symptoms similar to those characterised as chronic fatigue syndrome (CFS), which have therefore been described as CFS-like symptoms. The aetiology and pathophysiology of CFS is largely unknown, but changes in the immune system have been described and autoimmune mechanisms are suspected to play a role [17–19]. Some studies report that CFS is often precipitated by an infection [20–24]. Also, vaccinations have been proposed to be involved in the onset of the pathophysiological process of CFS [25,26]. However, no association between vaccination and CFS has been detected in epidemiological studies [8,25,26]. National health authorities generally recommend to postpone a vaccination in case of acute infections with fever, mainly because of difficulties in separating potential vaccine reactions from symptoms due to the infection [27–29]. However, no specific recommendation is given for an already treated or recent infection [27–29]. We hypothesised that an infection in temporal proximity to HPV vaccination could precipitate the reported CFS-like symptoms following vaccination. Thus, the objective of this study was to examine the association between infection in temporal proximity to HPV vaccination and referral to an HPV centre because of suspected adverse effects.

## Methods

### Setting and data sources

The study was a nationwide Danish register-based cohort study. In Denmark, every citizen is registered with a unique 10-digit civil personal registration (CPR) number, and we used this to link information at the individual level between the following national registries. *The Danish National Patient Registry*, which contains nationwide information on primary and secondary diagnoses associated with each inpatient, outpatient, and emergency room visit according to the International Classification of Diseases, 10th version (ICD-10) [30]. *The Danish Register of Medicinal Product Statistics*, which contains information on redeemed prescriptions of medication in community pharmacies. The information includes type and product name of medication [31,32]. *The Danish National Health Insurance Service Registry*, which contains information on contacts and specific services provided by the general practitioner (GP). The register is a reimbursement register and provides no information on reasons for contact, diagnosis, or test results [27,28]. *Statistics Denmark*, which contains information on population statistics including socioeconomic variables. *Electronic Patient Journals*, to identify females referred to an HPV centre for suspected adverse effects. There is no study protocol available for publication.

### Study population

The study population included all HPV-vaccinated females living in Denmark, born between 1974 and 2006, and vaccinated in the period between January 1, 2006 and December 31, 2017. Females who received the first HPV vaccination at the GP were identified in the Danish National Health Insurance Service Registry, in which the week of HPV vaccination is registered (service codes 8328, 8329, 8330 or 8334, 8335, and 8336). In addition, females who redeemed a first prescription of a HPV vaccine (4-valent, 2-valent, or 9-valent) at a community pharmacy were identified in the Danish Register of Medicinal Product Statistics (Anatomic Therapeutic Chemical (ATC) codes J07BM01, J07MB02, or J07BM03). The HPV vaccination date was a random allocated date in the week of receiving the first HPV vaccination at the GP or the date of redeeming the first HPV vaccine prescription at the pharmacy. If a female was registered with both a redemption of the HPV vaccine and a service code of HPV vaccination at the GP within a period of 14 days, we used the first date registered.

### Exposure

The exposure of interest was having an infection in the period from 1 month before until 1 month after the first HPV vaccination date. Presence of infection was defined by the following markers: (1) admission date for an inpatient or outpatient hospital contact associated with a primary (first-listed) discharge diagnosis of infection (specific ICD-10 codes in S1 Table); (2) date of redeeming at least one prescription of an anti-infective medication at a pharmacy (specific ATC codes in S2 and S3 Tables); and (3) date of a rapid streptococcal test (service code 7109) used to assist in the diagnosis of suspected pharyngitis at the GP. We categorised and included each female at the date of the most severe infection category ± 30 days around the HPV vaccination, with severity going from hospital-treated infection (most severe), through redemption of anti-infective medication only, and to rapid streptococcal test for pharyngitis without anti-infectives or hospital contact (least severe). In subanalyses, we further categorised anti-infective medications according to type as an indication of likely pathogens treated: bacterial, viral, fungal infection, and multiple infections (specific ATC codes in S2 Table), and according to likely site of the treated infection, e.g., urinary or upper respiratory tract (specific ATC codes in S3 Table). In a subanalysis, we excluded prescriptions of tetracycline, as these can be prescribed for several conditions including treatment of severe acne.

### Outcome

Females could be referred to any of the HPV centres, established in 2015, by their GP or by a medical specialist. The criteria for referral were unspecific or unexplained symptoms occurring in temporal relation to HPV vaccination. The outcome in this study was referral to one of these centres at some point in the period from July 1, 2015 until December 31, 2017.

### Confounding factors

Variables that may potentially confound the associations by being associated with both risk of infection and risk of later suspected adverse vaccine effects were identified a priori. Information related to social position in terms of maternal education and socioeconomic status of the family was obtained from Statistics Denmark for the year before first HPV vaccination [33]. We obtained information on chronic somatic conditions from the Danish National Patient Registry. Information on psychiatric conditions and asthma was obtained from the Danish National Patient Registry as well as from the Danish Register of Medicinal Product Statistics (see specific ICD-10 codes and ATC codes in S1 Text) [28,29]. Information on the somatic and psychiatric conditions were obtained in a period of 5 years before the date of first HPV vaccination. Finally, we accounted for age and calendar year as use of antibiotics decreased from 2008 to 2017 in all young age groups in Denmark [34].

### Statistical analyses

Characteristics of the study population according to infection in temporal proximity to HPV vaccination status are presented using number and percentages. Also, we graphically present the age distribution of the HPV vaccinated females according to year of vaccination as several catch-up programmes including older girls and young women have taken place in different years. All models were defined prior to conducting the study. We used simple and multivariable logistic regression to estimate the crude and adjusted odds ratio (OR) with 95% confidence intervals (CIs) as a measure of the association between each specific definition of infection and referral to an HPV centre. In a sensitivity analysis, log binomial regression analysis was applied instead. The associations were estimated both overall and stratified on age at vaccination (±18 years) and year of vaccination (2006 to 2012 and 2013 to 2017), respectively. All analyses were adjusted for the following variables: year of vaccination (2006 to 2008, 2009 to 2011, 2012 to 2013, 2014 to 2015, 2016 to 2017), age at vaccination (11 to 14, 15 to 17, 18 to 24, ≥25 years old), maternal educational level (PhD, higher education, medium-length education, basic education, high school or vocational, and primary school), socioeconomic status of the family (owner of business, chief executive or employee with high income, employee with middle income, employee with low income, employee with unspecified income, unemployed, and pensioner), as well as chronic somatic and psychiatric conditions in the 5 years prior to the first vaccine registration (yes/no). Since referral to an HPV centre is rare and we have virtually complete follow-up in the study period, the calculated ORs in the cohort study were interpreted as risk ratios.

### Case-crossover and case time-control analyses

**Fig 1:**
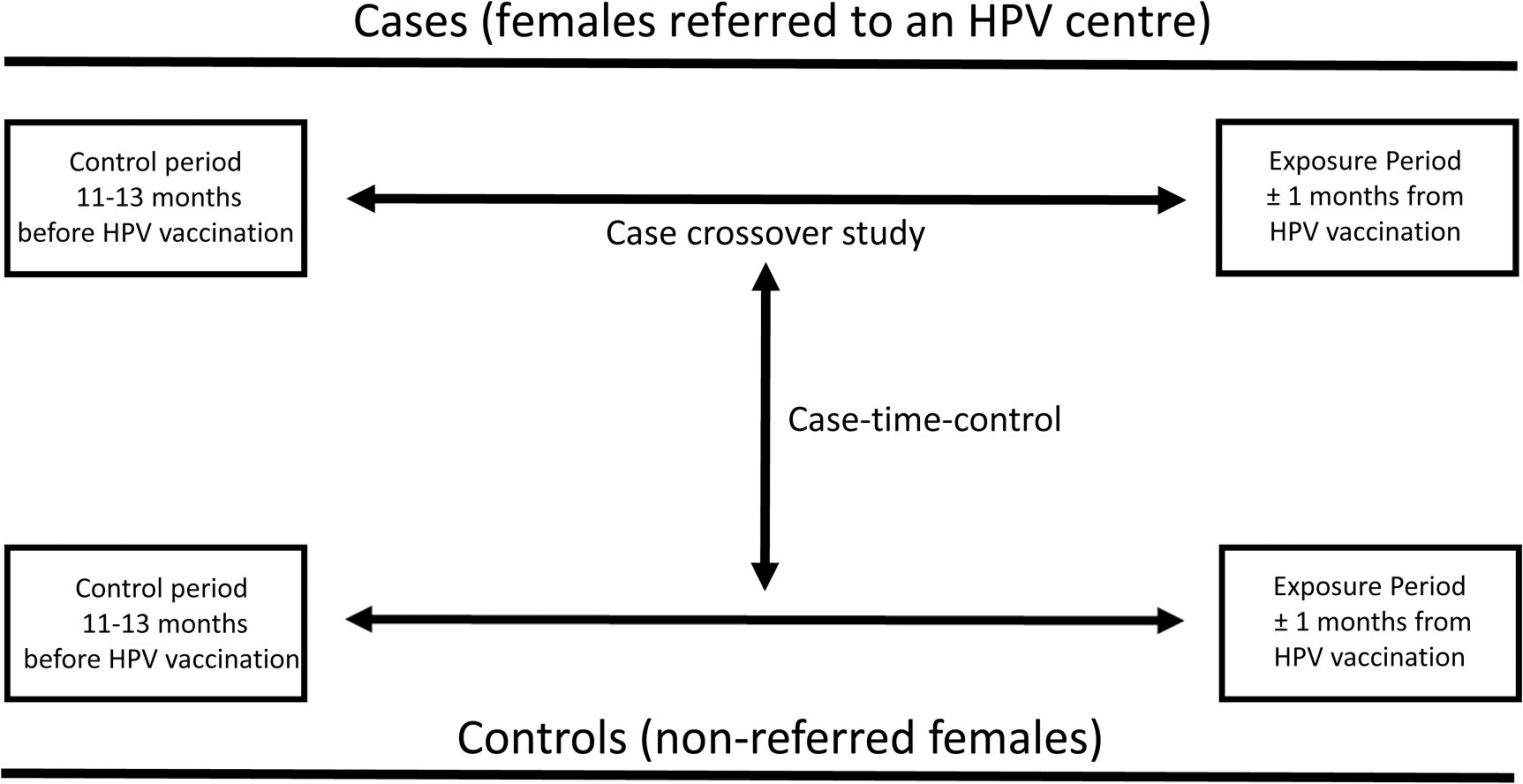
Case-crossover and case time-control design (inspired by Hernández-Díaz and colleagues [33]).

In order to understand whether unmeasured confounding could explain potential associations between infections in temporal proximity to vaccination and referral to an HPV centre, we performed a case-crossover and a case time-control design (Fig 1) [35,36]. In a first step, we used a case-crossover design, which only includes cases (all females referred to an HPV centre) and uses cases as their own controls (using different exposure periods). Thus, this design can effectively control for all personal time-stable factors. For each case, each type of infection was ascertained for the same “risk-window period” as the main analysis (±1 month of vaccination) and a “control-window period” of the same length the year before (11 to 13 months before vaccination). Exposure frequencies from the risk-window period and the control-window period were compared by calculating a matched OR. The case-crossover OR (OR_cases_) was estimated as the ratio of the number of cases exposed only in the risk-window period to the number of cases exposed only during the control-window period (i.e., the ratio of discordant pairs). In a second step, we used a case time-control design in order to control for temporal changes in infections from 1 year to the other. To do that, we estimated the matched OR for controls (all nonreferred females) as we did for cases: For each control, exposure frequencies from risk-window period and the control-window period were compared through an OR (OR_controls_). The case time-control OR was estimated as OR_adjusted_ = OR_cases_ / OR_controls_. All *p*-values related to specific estimates in the statistical analyses were estimated by Wald tests.

All statistical analyses were carried out using STATA 13 statistical software.

This study is reported as per the RECORD-PE guideline (S1 Checklist).

## Results

**Fig 2:**
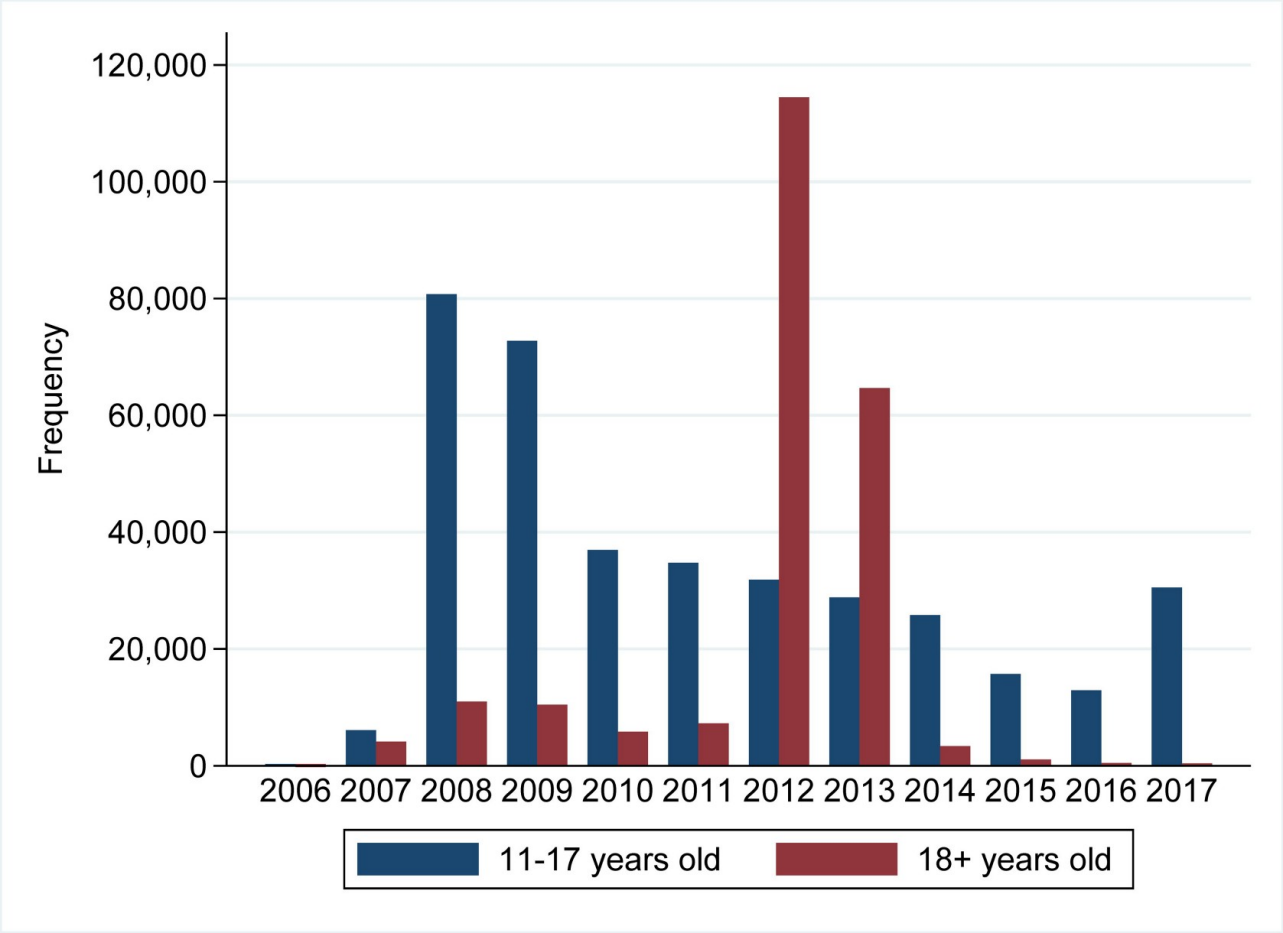
Age distribution at the time of first HPV vaccination according to calendar year.

The study population consisted of 600,400 HPV-vaccinated females aged 11 to 44 years at the time of first HPV vaccination. Fig 2 shows the age distribution according to year of vaccination. From 2006, the HPV vaccine was available as a self-paid vaccination. In 2008, a start-up programme was initiated, and the HPV vaccine was included in the national childhood programme in 2009. In addition, until 2016, several catch-up programmes targeting females aged 15 or older were carried out. The largest catch-up programme was completed in 2012 to 2013 where 239,797 (39.9% of all the HPV-vaccinated females) were vaccinated and 179,134 of these (74.7%) were aged 18 or older (Table 1 and Fig 2). The characteristics of the study population are presented in Table 1. Within 1 month before or after HPV vaccination, 2,608 (0.4%) females had a hospital-treated infection, 44,220 (7.4%) had redeemed a prescription of anti-infective medication without hospital treatment, and 11,533 (1.9%) had a rapid streptococcal test for pharyngitis infection at the GP with neither redemption anti-infective medication nor hospital treatment. Overall, compared to females without infection, infected females more likely suffered from asthma, lived in families where the parents either had low income, were pensioners or unemployed, and tended to have mothers with shorter education (high school or less). In addition, females with hospital-treated infections or females who redeemed anti-infective treatment in temporal proximity to HPV vaccination were more likely to have received the first HPV vaccination in the period from 2012 throughout 2013 and were generally older (18+ years). Finally, females with hospital-treated infections or females who redeemed anti-infective treatment were more likely to suffer from chronic somatic conditions and psychiatric conditions (Table 1).

**Table 1:** Characteristics of the study population by infection in temporal proximity to HPV vaccination.

	No infection	Infection stratified by severity of infection		
	n (%)	Hospital-treated infection n (%)	Redeemed anti-infective prescription n (%)	Rapid streptococcal test at the GP n (%)
N 600,400	542,039 (90.3)*	2,608 (0.4)*	44,220 (7.4)*	11,533 (1.9)*
**Year of first HPV vaccination**				
2006–2008**	92,244 (17.0)	360 (13.8)	7,111 (16.1)	2,618 (12.7)
2009–2011	154,190 (28.4)	648 (24.8)	9,492 (21.5)	3,647 (31.6)
2012–2013***	210,084 (38.8)	1,215 (46.6)	24,719 (55.9)	3,779 (32.8)
2014–2015	43,238 (8.0)	227 (8.7)	1,662 (3.8)	820 (7.1)
2016–2017	42,283 (7.8)	158 (6.1)	1,236 (2.8)	669 (5.8)
**Age at first HPV vaccination**				
11–14 years	297,822 (54.9)	704 (27.0)	10,530 (23.8)	6,163 (53.4)
15–17 years	55,286 (10.2)	215 (8.2)	4,551 (10.3)	1,864 (16.2)
18–25 years	123,720 (22.8)	1,157 (44.4)	19,120 (43.3)	2,430 (21.1)
26+ years	65,211 (12.0)	532 (20.4)	10,019 (22.7)	1,076 (9.3)
**Socioeconomic position of the family**				
Owner of business	31,198 (6.0)	125 (5.1)	2,446 (5.8)	616 (5.5)
Chief executive or employee with high income	116,196 (22.2)	495 (20.1)	8,168 (19.4)	2,126 (19.0)
Employee with middle income	95,706 (18.3)	415 (16.9)	7,341 (17.5)	2,022 (18.0)
Employee with low income	142,086 (27.2)	687 (27.9)	11,670 (27.8)	3,253 (29.0)
Employee unspecified income	75,762 (14.5)	349 (14.2)	6,131 (14.6)	1,751 (15.6)
Pensioners	26,740 (5.1)	199 (8.1)	3,426 (8.2)	585 (5.2)
Unemployed	32,057 (6.1)	173 (7.1)	2,515 (6.0)	798 (7.1)
Other	3,487 (0.7)	15 (0.6)	302 (0.7)	67 (0.6)
Missing	18,807	150	2,221	315
**Maternal education**				
PhD or higher education	39,815 (7.6)	160 (6.5)	2,200 (5.2)	599 (5.3)
Middle education	128,242 (24.6)	534 (21.7)	9,746 (23.2)	2,616 (23.3)
Basic education	28,764 (5.5)	127 (5.2)	2,058 (4.9)	534 (4.8)
High school or vocational	219,380 (42.0)	1,048 (42.7)	17,724 (42.2)	4,897 (43.7)
Primary school	97,732 (18.7)	549 (22.3)	9,638 (22.9)	2,387 (21.3)
Unknown	8,351 (1.6)	38 (1.5)	672 (1.6)	172 (1.5)
Missing	19,755	152	2,182	328
**Chronic somatic condition before first HPV vaccination**				
Yes	25,254 (4.7)	435 (16.7)	3,966 (9.0)	685 (5.9)
**Asthma before first HPV vaccination**				
Yes	64,103 (11.8)	462 (17.7)	7,412 (16.8)	1,944 (16.9)
**Psychiatric condition before first HPV vaccination**				
Yes	38,360 (7.1)	462 (17.7)	7,063 (16.0)	948 (8.2)

*Row %.

**Start-up HPV vaccination programme including females born in the period 1993–1995.

***Large catch-up HPV vaccination programme for females born in the period 1985–1992.

GP, general practitioner; HPV, human papilloma virus.

Referral to an HPV centre was rare. A total of 1,755 (0.3%) of the HPV-vaccinated females were referred to an HPV centre during the study period (Table 2). Overall, females with infection in temporal proximity to HPV vaccination had higher risk of referral to an HPV centre compared to females without an infection. The strongest association was detected among females who had a hospital-treated infection (OR 2.75, 95% CI 1.72 to 4.40; *P* < 0.001) compared to females with no infection (Table 2). In addition, redemption of an anti-infective prescription and having a rapid streptococcal test at the GP were also associated with a higher risk of referral compared to no infection (OR 1.56, 95% CI 1.33 to 1.83; *P* < 0.001 and OR 1.45, 96% CI 1.10 to 1.93; *P* = 0.010), respectively (Table 2). Exclusion of prescriptions for tetracycline from the anti-infective prescriptions did not change the result (OR 1.57, 95% 1.33 to 1.85; *P* < 0.001). After stratifying on age at first HPV vaccination (±18 years), the higher risk of referral in females with an infection in temporal proximity to HPV vaccination was detected in both age groups. However, point estimates tended to be larger for females 18+ years old. No difference was noticed when stratifying on year of vaccination (2006 to 2012/2013 to 2017) (S4 Table).

**Table 2:** Association between infection in temporal proximity to first HPV vaccination (±1 month) and later referral to an HPV centre for suspected adverse vaccine effects.

	Total (N)	Females referred to an HPV centre (n)	Females referred to an HPV centre per 10,000	Unadjusted OR (95% CI)		Adjusted OR* (95% CI)					
				Overall				Stratified on age at vaccination			
	600,400	1,755			*p-*value^†^		*p-*value^†^	<18 years	*p-*value^†^	≥18 years	*p-*value^†^
No infection	542,039	1,505	27.8	-ref-		-ref-		-ref-		-ref-	
Hospital contact due to infection	2,608	18	69.0	2.50 (1.57;3.98)	<0.001	2.75 (1.72;4.40)	<0.001	2.55 (1.21;5.39)	0.014	2.85 (1.56;5.23)	0.001
Redeemed anti-infective prescription	44,220	181	40.9	1.48 (1.26;1.72)	<0.001	1.56 (1.33;1.83)	<0.001	1.40 (1.10;1.78)	0.007	1.71 (1.37;2.12)	<0.001
Rapid streptococcal test by the GP	11,533	51	44.2	1.60 (1.21;2:11)	0.001	1.45 (1.10;1.93)	0.010	1.38 (0.99;1.91)	0.056	1.72 (0.99;2.99)	0.057

*Adjusted for age at vaccination, year of vaccination, maternal education, socioeconomic position of the family, and chronic somatic conditions, asthma, and psychiatric conditions.

^**†**^Wald test.

CI, confidence interval; GP, general practitioner; HPV, human papilloma virus; OR, odds ratio.

The most common type of redeemed anti-infective medication according to likely treated pathogen was anti-bacterial (*n =* 34,863; 79% of all anti-infective prescriptions), while the most likely site of the treated infection was the respiratory tract (18,663; 42%) (Table 3). Overall, infections according to most types of anti-infective medication were associated with increased risk of later referral to an HPV centre; however, no substantial differences were detected between the likely treated pathogen or likely site of infection and later referral to an HPV centre. Additionally, classification of likely targeted site of the infection was not possible for 11,693 (26%) of the redeemed prescriptions as the anti-infective medication could be targeted for infections in several different sites (Table 3). The sensitivity analyses using log binomial regression analysis yielded almost identical results (S5 and S6 Tables).

**Table 3:** Association between infection according to type of redeemed anti-infective medication in temporal proximity to first HPV vaccination (±1 month) and later referral to an HPV centre for suspected adverse vaccine effects.

		Total (N) 586,259*	Females referred to an HPV centre (n) 1,686	Females referred to an HPV centre n per 10,000	Unadjusted OR (95% CI)	*p-*value^†^	Adjusted** OR (95% CI)	*p-*value^†^
No infection		542,039	1,505	27.7	-ref-		-ref-	
	**Type of anti-infective medication as an indication of likely pathogens treated**							
Antibacterial medication		34,863	147	42.2	1.52 (1.28;1.80)	<0.001	1.55 (1.30;1.85)	<0.001
Antiviral medication		2,715	8	29.5	1.06 (0.53;2.13)	0.867	1.29 (0.64;2.59)	0.475
Antimycotic medication		4,484	14	31.2	1.12 (0.66;1.91)	0.662	1.44 (0.85;2.45)	0.177
Multiple types of medication		2,158	12	55.6	2.01 (1.14;3.56)	0.016	2.41 (1.35;4.28)	0.003
	**Type of anti-infective medication as an indication of likely site of the treated infection**							
Respiratory tract infection		18,663	91	48.8	1.72 (1.39;2.13)	<0.001	1.66 (1.34;2.07)	<0.001
Urinary tract infection		8,282	37	44.7	1.48 (1.05;2.08)	0.024	1.60 (1.13;2.27)	0.008
Skin/skin mycosis or soft tissue bacterial infection		2,522	5	19.8	0.72 (0.30;1.73)	0.458	0.72 (0.30;1.73)	0.464
Herpes simplex or varicella-zoster infection		3,007	10	33.3	1.19 (0.64;2.23)	0.576	1.45 (0.78;2.71)	0.224
Infection no possible categorisation**		11,693	43	36.8	1.32 (0.97;1.79)	0.073	1.59 (1.17;2.17)	0.003

*Females with hospital-treated infections or RST at the GP were excluded from the analyses.

**Adjusted for age at vaccination, year of vaccination, maternal education, socioeconomic position of the family, chronic somatic conditions, asthma, and psychiatric conditions.

***Categorisation not possible as the medication are prescribed for infection in different organ systems.

^**†**^Wald test.

CI, confidence interval; GP, general practitioner; HPV, human papilloma virus; OR, odds ratio; RST, rapid streptococcal test.

### Case-crossover and case time-control analyses

Although controls were more likely to experience a hospital-treated infection or anti-infective prescription in temporal proximity to vaccination than in the year before (indicating moderate trends of exposure to an infection), cases were even more likely to have an infection around vaccination than in the year before (Table 4). Thus, after adjustment for the time trend, we observed an association between anti-infective medication redeemed around the time of HPV vaccination and later referral to an HPV centre (OR 1.40, 95% CI 1.08 to 1.81; *P* = 0.011). Although the association with referral to an HPV centre was based on a small number of discordant pairs and not statistically significant for a hospital-treated infection happening around the time of HPV vaccination, the point estimate was larger than for those with anti-infective prescriptions (OR 1.84, 95% CI 0.74 to 4.56; *P* = 0.189). On the contrary, the association for rapid streptococcal test at the GP and referral to an HPV centre due to suspected adverse effects was weaker (OR 1.11, 95% CI 0.73 to 1.70; *P* = 0.718).

**Table 4:** Case-crossover and case time-control analyses of the association between infection in temporal proximity to HPV vaccination (±1 month) and referral to an HPV centre (reference period 11–13 months before HPV vaccination).

	Referred females n/discordant pairs	Referred females OR (95%)	Nonreferred females (time trend) n/discordant pairs	Nonreferred females (time trend) OR (95% CI)	*p*-value^†^	Referred females OR (95% CI) adjusted for time trend	*p*-value^†^
Hospital-treated infections	14/7	2.00 (0.81;4.96)	2,004/1,841	1.09 (1.02;1.16)	0.009	1.84 (0.74;4.56)	0.189
Redeemed anti-infective prescription	143/95	1.51 (1.16;1.95)	35,025/32,519	1.08 (1.06;1.09)	<0.001	1.40 (1.08;1.81)	0.011
Rapid streptococcal test	47/43	1.09 (0.72;1.65)	9,756/9,632	1.01 (0.98;1.04)	0.373	1.11 (0.73;1.70)	0.718

^**†**^Wald test.

CI, confidence interval; HPV, human papilloma virus; OR, odds ratio.

## Discussion

In this nationwide population-based cohort study, which included designs to control for unmeasured confounding, we observed that a hospital-treated infection or redemption of anti-infective treatment in temporal proximity to HPV vaccination was associated with a higher risk of referral to an HPV centre because of suspected adverse effects. The infected females had a higher risk of referral to an HPV centre regardless of the type of redeemed anti-infective treatment.

### Strengths and limitations

A strength of our study is the population-based design with virtually complete follow-up, which limits the risk of selection bias. Furthermore, in Denmark, there is free access to both primary and secondary healthcare, and all information in the registries on diagnoses, prescriptions, and special services were collected prospectively. Generally, the information from The Danish National Patient Registry and Danish Register of Medicinal Product Statistics are of high quality, and the validity of diagnoses and medication is high [30–32], and we therefore consider the risk of information bias for these exposures low. Information on rapid streptococcal test from the Danish National Health Insurance Service Registry does not include any information on diagnoses or positive/negative test results and is therefore mainly a measure of indication/suspicion of an infection. The GPs’ decision to provide a test or not in case of infection symptoms in temporal proximity to HPV vaccination is most likely unrelated to later referral to an HPV centre, but the females’ individual healthcare attendance behaviour might influence the likelihood of having a test for infection, and some infections might not be captured. Also, females with later symptoms who could recall other health effects (e.g., infection) happening around the time of HPV vaccination might in theory be less likely to be referred, as they had an alternative explanation for their symptoms. However, this potential detection bias cannot explain the positive associations found in the present study. Another strength in our study is that we were able to adjust for a range of confounders, including prior somatic conditions, asthma, and psychiatric conditions, based on highly valid information from registries. Additionally, we conducted a case-crossover and a case time-control analyses in order to control for unmeasured/uncontrolled confounding including potential differences in healthcare attendance pattern. The results of the case-crossover and case time-control analyses supported our findings for females with hospital-treated infections and for females who had redeemed anti-infective medication. Conversely, the analysis did not support the finding for females tested for pharyngitis at the GP. A possible explanation for this could be that having a test for infection at the GP is more likely influenced by healthcare attendance pattern than a hospital-treated infection or receiving medication for an infection. This explanation is supported by Danish case–control studies reporting differences in healthcare attendance prior to HPV vaccination between females referred to an HPV centre and nonreferred females [37–39]. The associations detected for the treated infections using both study designs support the robustness of these findings. A limitation of our study is that the HPV centres did not open until June 2015. We could therefore not include time to referral in our analyses. Also, some females experiencing adverse effects well before this date might have had other diagnoses and treatment and consequently never been referred to an HPV centre. To assess any bias from possible incomplete outcome registration early in the study period, we stratified the main analysis on calendar year of first HPV vaccination (2006 to 2012 and 2013 to 2017; see S4 Table). No major differences in relative risk estimates across the periods were observed.

### Comparison with other studies

To our knowledge, no other studies have investigated the association between infections in temporal proximity to HPV vaccination and suspected adverse vaccine effects. However, some studies have investigated the association between infection or vaccination and the CFS diagnosis. In a Norwegian nationwide register-based follow-up study, the researchers found that CFS was associated with pandemic influenza infection, but not with a pandemic influenza vaccine [26]. Correspondingly, in a case–control study, no association between vaccination against meningococcal disease in teenagers and CFS was observed, whereas the authors found an association between infectious mononucleosis and CFS [25]. Finally, no association between HPV vaccination and CFS was detected in another Norwegian nationwide register-based follow-up study [8]. These studies support our hypothesis of infection in temporal proximity to HPV vaccination as a potential trigger of the CFS-like symptoms. As we only included HPV-vaccinated females in our study, we are not able to differentiate possible effects of an infection in itself from a potential interaction between an infection and the HPV vaccination. Furthermore, we are not able to differentiate effects of the infection per se from the treatment given for the infection. Future studies should aim at investigating the specific mechanisms further.

## Implications

In clinical guidelines, it is recommended to reschedule a vaccination in febrile persons for 2 reasons. First of all, the vaccination might not be effective, and, secondly, it is difficult to differentiate between potential vaccine reactions and signs of illness [27]. However, treated infections are not described as a reason for delay of vaccination [27]. In our study, we observed that infection in temporal proximity to HPV vaccination was associated with a higher risk of referral to an HPV centre because of suspected adverse effects than noninfected females. To our knowledge, this is the first study to investigate this association. If these results can be replicated in other study populations, health authorities should consider primarily scheduling school-based HPV vaccination programmes and individual vaccinations in the months where the risk of infections is low, in order to lower the risk of misinterpretation of symptoms. However, potential consequences of HPV vaccine postponement should also be taken into account. It is important to emphasise that the absolute risk of referral to an HPV centre was low and that having an infection in temporal proximity to first HPV vaccination only explained a limited proportion of the total number of referrals (14.5% of the referred females had an infection in temporal proximity to first HPV vaccination). However, preventing erroneous suspicion of suspected adverse effects following HPV vaccination is not only of interest for the individual; it is also important in a public health perspective in terms of maintaining public confidence towards the HPV vaccination programme.

## Conclusions

Infection in temporal proximity to HPV vaccination was associated with a higher risk of referral to an HPV centre because of suspected adverse vaccine effects. The infection could potentially be a trigger of the CFS-like symptoms in a subset of the referred females. Further studies are needed to detect whether interaction between infection and HPV vaccination plays a role.

## Supporting information

S1 Table List of ICD-10 codes for hospital treated infections. (DOCX)

S2 Table List of anti-infective products (ATC-Codes). (DOCX)

S3 Table Likely site of infection for which redeemed anti-infectives are used. (DOCX)

S4 Table Association between infection in temporal proximity to first HPV vaccination (±1 month) and later referral to an HPV centre for suspected adverse vaccine effects stratified on year of first HPV vaccination (2006–2012/2013–2017). (DOCX)

S5 Table Association between infection in temporal proximity to first HPV vaccination (±1 month) and later referral to an HPV centre for suspected adverse vaccine effects (RR, 95% CI). (DOCX)

S6 Table. Association between infection according to type of redeemed anti-infective medication in temporal proximity to first HPV vaccination (±1 month) and later referral to an HPV centre for suspected adverse vaccine effects (RR, 95% CI) (DOCX)

S1 Text ICD-10 codes used to identify somatic and psychiatric conditions. (DOCX)

S1 Checklist The RECORD statement for pharmacoepidemiology (RECORD-PE) checklist of items, extended from the STROBE and RECORD statements, which should be reported in noninterventional pharmacoepidemiological studies using routinely collected health data. (DOCX)

## Abbreviations

ATC: Anatomic Therapeutic Chemical
CFS: chronic fatigue syndrome
CI: confidence interval
CPR: civil personal registration
GP: general practitioner
HPV: human papilloma virus
OR: odds ratio
