# Supplementary material for: Infections in temporal proximity to HPV vaccination and adverse effects following vaccination in Denmark: A nationwide register-based cohort study and case-crossover analysis
Source: PLoS Med. 2021 Sep 8;18(9):e1003768. doi: 10.1371/journal.pmed.1003768 (PMC8457493; doi:10.1371/journal.pmed.1003768)
Supplement: S1 Table — (DOCX) [file pmed.1003768.s001.docx]

| **Supplementary Table 1 List of ICD-10 codes for hospital treated infections** |
| --- |
| **Hospital treated infections (inpatient and outpatient) A-diagnosis*** |
| **ICD-10 code**  Entire A and B chapter  G00–G02, G04–G07  H00, H01.0, H03.0–H03.1, H04.0, H04.3, H05.0, H06.1, H10, H13.1, H19.1–19.2, H22.0, H32.0, H44.0, H60.0–H60.3, H62.0–H62.4, H65.0, H66, H67.0–H67.1, H70, H73.0, H75.0, H94.0  I00–I02, I30.1, I32.0–I32.1, I33.0, I38, I39.8, I40.0, I41.0–I41.2, I43.0, I52.0–I52.1, I68.1, I98.1, J00–J06, J09–J18, J20–J22, J34.0, J36, J39.0–J39.1, J44.0, J85–J86  K04.6, K04.7, K05.2, K11.2–K11.3, K12.2, K23.0–K23.1, K35, K37, K57.0, K57.2, K57.4, K57.8, K61, K63.0, K65.0, K67, K75.0–K75.1, K77.0, K80.0, K80.3–K80.4, K81.0, K83.0, K85.9, K93.0–K93.1  L00–L03, L05–L08  M00–M03, M46.3, M46.5, M49.0–M49.3, M60.0, M63.0–M63.2, M65.0–M65.1, M68.0, M71.0–M71.1, M86, M90.0–M90.2  N10–N12, N15.1, N16.0, N29.1, N30.0, N33.0, N34.0–N34.1, N39.0, N41, N43.1, N45, N48.1–N48.2, N49, N51, N61, N70–77 |
| *Primary diagnosis associated with inpatient and outpatient visit according to the International Classification of Diseases 10^th^ version (ICD-10). |
