# Supplementary material for: Infections in temporal proximity to HPV vaccination and adverse effects following vaccination in Denmark: A nationwide register-based cohort study and case-crossover analysis
Source: PLoS Med. 2021 Sep 8;18(9):e1003768. doi: 10.1371/journal.pmed.1003768 (PMC8457493; doi:10.1371/journal.pmed.1003768)
Supplement: S2 Table — (DOCX) [file pmed.1003768.s002.docx]

| **Supplementary Table 2 List of anti-infective products (ATC-Codes)** | |
| --- | --- |
| **Infection** | **Pharmaceutical product (ATC-Code)** |
| Anti-bacterials for systemic use | J01 |
| Anti-virals for systemic use | J05 |
| Anti-mycotics for systemic use | J02 |
