# Supplementary material for: Infections in temporal proximity to HPV vaccination and adverse effects following vaccination in Denmark: A nationwide register-based cohort study and case-crossover analysis
Source: PLoS Med. 2021 Sep 8;18(9):e1003768. doi: 10.1371/journal.pmed.1003768 (PMC8457493; doi:10.1371/journal.pmed.1003768)
Supplement: S3 Table — (DOCX) [file pmed.1003768.s003.docx]

| **Supplementary Table 3 Likely site of infection for which redeemed anti-infectives are used** | |
| --- | --- |
| **Likely site of infection for which the redeemed anti-infectives are used** | **Pharmaceutical product (ATC-Code)** |
| Respiratory tract infection | Pivampicillin (J01CA02)  Amoxicillin (J01CA04)  Phenoxymethylpenicillin (J01CE02)  Amoxicillin and enzyme inhibitor (J01CR02)  Erythromycin (J01FA01)  Roxithromycin (J01FA06)  Clarithromycin (J01FA09) |
| Urinary tract infections | Pivmecillinam (J01CA08)  Trimethoprim (J01EA01)  Sulfamethizole (J01EB02)  Sulfamethoxazole and Trimethoprim (J01EE01)  Nitrofurantoin (J01XE01) |
| Skin or soft tissue infections | Dicloxacillin (J01CF01)  Flucloxacillin (J01CF05) |
| Skin mycosis | Ketoconazole (J02AB02) |
| Herpes simplex or varicella-zoster infection | Aciclovir (J05AB01)  Famciclovir (J05AB09) |
| Redeemed anti-infective medication, where classification of likely site of infection were not possible. | Doxycycline (J01AA02)  Lymecycline (J01AA04)  Oxytetracycline (J01AA06)  Tetracycline (J01AA07)  Ampicillin (J01CA01)  Benzylpencillin (J01CE01)  Cefalexin (J01DB01)  Cefuroxime (J01DC02)  Ceftazidme (J01DD02)  Azithromycin (J01FA10)  Clindamycin (J01FF01)  Tobramycin (J01GB01)  Ofloxacin (J01MA01)  Ciprofloxacin (J01MA02)  Moxifloxacin (J01MA14)  Colistin (J01XB01)  Fusidic acid (J01XC01)  Fluconazole (J02AC01)  Itraconazole (J02AC02)  Rifampicin (J04AB02)  Dapsone (J04BA02)  Valaciclovir (J05AB11)  Oseltamivir (J05AH02) |
