# Supplementary material for: Infections in temporal proximity to HPV vaccination and adverse effects following vaccination in Denmark: A nationwide register-based cohort study and case-crossover analysis
Source: PLoS Med. 2021 Sep 8;18(9):e1003768. doi: 10.1371/journal.pmed.1003768 (PMC8457493; doi:10.1371/journal.pmed.1003768)
Supplement: S4 Table — (DOCX) [file pmed.1003768.s004.docx]

| **Supplementary Table 4 Association between infection in temporal proximity to first HPV vaccination (**± **one month) and later referral to an HPV-centre for suspected adverse vaccine effects stratified on year of first HPV vaccination (2006-2012/2013-2017)** | | | | | | | |
| --- | --- | --- | --- | --- | --- | --- | --- |
|  | Total (N) | Females referred to an HPV-centre (n) | Females referred to an HPV-centre  per 10,000 | Adjusted OR* (95% CI) | | | |
|  |  |  |  | Year of first HPV vaccination | | | |
|  | 600,400 | 1,755 |  | 2006-2012 | *p-value****†*** | 2013-2017 | *p-value****†*** |
| No infection | 542,039 | 1,505 | 27.8 | -ref- |  | -ref- |  |
| Hospital contact due to infection | 2,608 | 18 | 69.0 | 2.46 (1.39-4.37) | 0.002 | 3.61 (1.59-8.18) | 0.002 |
| Redemption of anti-infective prescription | 44,220 | 181 | 40.9 | 1.55 (1.30-1.86) | <0.001 | 1.59 (1.13-2.24) | 0.008 |
| Rapid Streptococcal Test by the GP | 11,533 | 51 | 44.2 | 1.43 (1.04-1.96) | 0.027 | 1.56 (0.83-2.93) | 0.168 |
| *Adjusted for age at vaccination, year of vaccination, maternal education, socioeconomic position of the family and chronic somatic conditions, asthma and psychiatric conditions.  † Walds test | | | | | | | |
