# Supplementary material for: Infections in temporal proximity to HPV vaccination and adverse effects following vaccination in Denmark: A nationwide register-based cohort study and case-crossover analysis
Source: PLoS Med. 2021 Sep 8;18(9):e1003768. doi: 10.1371/journal.pmed.1003768 (PMC8457493; doi:10.1371/journal.pmed.1003768)
Supplement: S5 Table — (DOCX) [file pmed.1003768.s005.docx]

| **Supplementary Table 5 Association between infection in temporal proximity to first HPV vaccination (**± **one month) and later referral to an HPV-centre for suspected adverse vaccine effects (RR, 95% CI)** | | | | | | | | | | | |
| --- | --- | --- | --- | --- | --- | --- | --- | --- | --- | --- | --- |
|  | Total (N) | Females referred to an HPV-centre (n) | Females referred to an HPV-centre  per 10,000 | Unadjusted RR (95% CI) | | Adjusted RR* (95% CI) | | | | | |
|  |  |  |  | Overall | | | | Stratified on age at vaccination | | | |
|  | 600,400 | 1,755 |  |  | | | | <18 years | | ≥18 years | |
| No infection | 542,039 | 1,505 | 27.8 | -ref- | p-value† | -ref- | p-value† | -ref- | p-value† | -ref- | p-value† |
| Hospital contact due to infection | 2,608 | 18 | 69.0 | 2.49 (1.56;3.95) | <0.001 | 2.73 (1.71;4.35) | <0.001 | 2.53 (1.21;5.31) | 0.014 | 2.84 (1.56;5.17) | 0.001 |
| Anti-infective prescription | 44,220 | 181 | 40.9 | 1.47 (1.26;1.72) | <0.001 | 1.56 (1.33;1.83) | <0.001 | 1.40 (1.10;1.78) | 0.007 | 1.70 (1.37;2.11) | <0.001 |
| Rapid Streptococcal Test by the GP | 11,533 | 51 | 44.2 | 1.59 (1.21;2:10) | 0.001 | 1.45 (1.10;1.92) | 0.010 | 1.38 (0.99;1.91) | 0.056 | 1.71 (0.99;2.98) | 0.056 |
| *Adjusted for age at vaccination, year of vaccination, maternal education, socioeconomic position of the family and chronic somatic conditions, asthma and psychiatric conditions.  † Walds test | | | | | | | | | | | |
