# Supplementary material for: Infections in temporal proximity to HPV vaccination and adverse effects following vaccination in Denmark: A nationwide register-based cohort study and case-crossover analysis
Source: PLoS Med. 2021 Sep 8;18(9):e1003768. doi: 10.1371/journal.pmed.1003768 (PMC8457493; doi:10.1371/journal.pmed.1003768)
Supplement: S6 Table. Association between infection according to type of redeemed anti-infective medication in temporal proximity to first HPV vaccination (±1 month) and later referral to an HPV centre for suspected adverse vaccine effects (RR, 95% CI) — (DOCX) [file pmed.1003768.s006.docx]

| **Supplementary Table 6 Association between infection according to type of redeemed anti-infective medication in temporal proximity to first HPV vaccination (**± **one month) and later referral to an HPV-centre for suspected adverse vaccine effects (RR, 95% CI)** | | | | | | | |
| --- | --- | --- | --- | --- | --- | --- | --- |
|  | Total (N)  586,259* | Females referred to an HPV-centre (n)  1,686 | Females referred to an HPV-centre n per 10.000 | Unadjusted RR  (95% CI) | | Adjusted** RR  (95% CI) | |
| No infection | 542,039 | 1,505 | 27.7 | -ref- | p-value† | -ref- | p-value† |
| **Type of anti-infective medication as an indication of likely pathogens treated** | | | | | | | |
| Anti-bacterial medication | 34,863 | 147 | 42.2 | 1.52 (1.28;1.80) | <0.001 | 1.55 (1.30;1.84) | <0.001 |
| Anti-viral medication | 2,715 | 8 | 29.5 | 1.06 (0.53;2.12) | 0.867 | 1.29 (0.64;2.58) | 0.476 |
| Anti-mycotic medication | 4,484 | 14 | 31.2 | 1.12 (0.66;1.90) | 0.662 | 1.44 (0.85;2.44) | 0.177 |
| Multiple types of medication | 2,158 | 12 | 55.6 | 2.01 (1.14;3.54) | 0.016 | 2.40 (1.35;4.24) | 0.003 |
| **Type of anti-infective medication as an indication of likely site of the treated infection** | | | | | | | |
| Respiratory tract infection | 18,663 | 91 | 48.8 | 1.72 (1.39;2.13) | <0.001 | 1.66 (1.33;2.06) | <0.001 |
| Urinary tract infection | 8,282 | 37 | 44.7 | 1.48 (1.05;2.08) | 0.024 | 1.60 (1.13;2.26) | 0.008 |
| Skin/skin mycosis or soft tissue bacterial infection | 2,522 | 5 | 19.8 | 0.72 (0.30;1.73) | 0.459 | 0.72 (0.30;1.73) | 0.465 |
| Herpes simplex or varicella-zoster infection | 3,007 | 10 | 33.3 | 1.19 (0.64;2.22) | 0.576 | 1.45 (0.78;2.70) | 0.244 |
| Infection no possible categorization** | 11,693 | 43 | 36.8 | 1.32 (0.98;1.79) | 0.073 | 1.59 (1.17;2.16) | 0.003 |
| *Females with hospital treated infections or RST test at the GP were excluded from the analyses  **Adjusted for age at vaccination, year of vaccination, maternal education, socioeconomic position of the family, chronic somatic conditions, asthma and psychiatric conditions.  ***Categorization not possible as the medication are prescribed for infection in different organ systems  † Walds test | | | | | | | |
