# Supplementary material for: Infections in temporal proximity to HPV vaccination and adverse effects following vaccination in Denmark: A nationwide register-based cohort study and case-crossover analysis
Source: PLoS Med. 2021 Sep 8;18(9):e1003768. doi: 10.1371/journal.pmed.1003768 (PMC8457493; doi:10.1371/journal.pmed.1003768)
Supplement: S1 Text — (DOCX) [file pmed.1003768.s007.docx]

**Supplementary text 1: ICD-10 codes used to identify somatic and psychiatric conditions**

Somatic conditions

From the Danish National Patient Registry the following ICD-10 codes were identified: DG40 (epilepsy), DM08 (juvenile arthritis), DN0-DN39 (renal disease), DE10-DE14 (diabetes mellitus), DK50-51 (Crohn disease and ulcerative colitis), DK900 (coeliac disease), DH54 (visual impairment), DH90-91 (hearing loss), DG80 (cerebral palsy), DQ05, (spina bifida), DG71 (muscular disease), DI* (Cardiovascular diseases), DJ45 (Asthmatic conditions). Furthermore, we obtained information on redemption of prescription medication for asthmatic conditions the National Patient Registry using the following ATC codes; R03A, R03B, R03C and R03D. Asthmatic conditions were defined as redemption of ≥2 prescriptions for β2-agonists or steroids (R03A, R03B and R03C), and ≥1 prescriptions for leukotriene receptor antagonists (R03DC).

Psychiatric conditions

From the Danish National Patient registry the following ICD-10 codes were identified DF20-29 (Schizophrenia, schizotypal and delusional disorders), DF30-39 (affective disorders), DF40-49 (Neurotic, stress-related and somatoform disorders), DF50 (eating disorder) and DF90 (Disturbance of activity and attention). Furthermore, we obtained information on redemption of prescription medication for psychiatric medication: N06A* (antidepressants), N05A* (antipsychotics), N06BA* (N05BA* (anxiolytics), N05BB* (anxiolytics), N03AX16 (anxiolytics) and N05BE01 (anxiolytics). The ATC codes N06AA (tricyclic antidepressants) and N06AX12 (bupropion) were excluded because of their frequent use for insomnia, smoking cessation and as pain medication.

|  |
| --- |
